# Platform for Analysis and Labeling of Medical Time Series

**DOI:** 10.3390/s20247302

**Published:** 2020-12-19

**Authors:** Andrejs Fedjajevs, Willemijn Groenendaal, Carlos Agell, Evelien Hermeling

**Affiliations:** Stichting Imec the Netherlands, 5656 AE Eindhoven, The Netherlands; willemijn.groenendaal@imec.nl (W.G.); carlos.agell@imec.nl (C.A.); evelien.hermeling@imec.nl (E.H.)

**Keywords:** annotation, peak detection, photoplethysmography signal, QRS detection, software tool, user interface

## Abstract

Reliable and diverse labeled reference data are essential for the development of high-quality processing algorithms for medical signals, such as electrocardiogram (ECG) and photoplethysmogram (PPG). Here, we present the Platform for Analysis and Labeling of Medical time Series (PALMS) designed in Python. Its graphical user interface (GUI) facilitates three main types of manual annotations—(1) fiducials, e.g., R-peaks of ECG; (2) events with an adjustable duration, e.g., arrhythmic episodes; and (3) signal quality, e.g., data parts corrupted by motion artifacts. All annotations can be attributed to the same signal simultaneously in an ergonomic and user-friendly manner. Configuration for different data and annotation types is straightforward and flexible in order to use a wide range of data sources and to address many different use cases. Above all, configuration of PALMS allows plugging-in existing algorithms to display outcomes of automated processing, such as automatic R-peak detection, and to manually correct them where needed. This enables fast annotation and can be used to further improve algorithms. The GUI is currently complemented by ECG and PPG algorithms that detect characteristic points with high accuracy. The ECG algorithm reached 99% on the MIT/BIH arrhythmia database. The PPG algorithm was validated on two public databases with an F1-score above 98%. The GUI and optional algorithms result in an advanced software tool that allows the creation of diverse reference sets for existing datasets.

## 1. Introduction

Recent developments in personalized medicine and wearable sensors have resulted in a great increase in time series datasets [1] being acquired in clinical and ambulatory settings over a variety of applications [2,3]. To fully exploit the data, automated feature extraction algorithms are in high demand. Training and validation of these algorithms requires large annotated datasets focusing on various scenarios. To allow fast and accurate annotation of these datasets a software tool is needed that meets the following requirements: it should (1) be flexible for different time series input data from various applications, (2) allow different types of annotation to be used and (3) allow customization to facilitate annotations. 

First, to be able to address a wide range of applications, the tool needs to accept and ensure similar functionality for many different data sources, e.g., respiratory, cardiac, or neurological signals. This implies no restrictions on input signal type, number of channels, and source of signals, as well as no predefined data structure. Such annotation tool should support loading of public, standardized, and unconventional datasets with multiple time-series signals. Related to that, it should be possible to (re)load the output of the tool again in itself as well as in standard programming languages.

Second, the tool should allow for different types of annotation. Biomedical signals are often annotated for specific event occurrence by their timestamp and/or amplitude, e.g., R-peaks in electrocardiogram (ECG) signal. In addition, signals are frequently annotated for quality evaluations, e.g., to label motion artifacts, or for labeling of fixed duration time segments, e.g., sleep apnea labeling in 30-s intervals. 

Another requirement is the need for a high-quality user experience. Manual labeling requires high concentration and is prone to errors [4,5]. Facilitating easy and smooth annotation could reduce the number of errors in the annotations. Therefore, a rapid and easy to operate graphical user interface (GUI) is required. The GUI needs to be able to load heavy datasets with many recordings. Browsing through the datasets should be implemented in an ergonomic manner, including intuitive zooming and scrolling and on-demand plotting of synchronized reference signals.

Preferably, the software should provide an interface for custom processing of the data. Plug-ins such as filtering or resampling operations allow flexible data explorations when dealing with generic data from various sources. Ultimately, if plug-ins themselves are more advanced algorithms, such as R-peak detection, working with the tool can be faster by enabling automatic labeling with subsequent manual adjustments and corrections. In this case, the GUI should clearly display pre-loaded or pre-detected annotations and provide a way to effectively delete or correct these.

Currently, several software solutions with manual annotation are available [6,7,8,9,10,11,12,13,14,15]. However, to the best of the authors’ knowledge none of these tools meet all requirements listed above. An often-encountered limitation is that they only work for one signal type—predominantly, ECG [6,7,8,9,10,11,12,13,14] or respiration [15]. Furthermore, the list of characteristic points is predefined: a user can annotate a single fiducial (R-peak) [10,14] or an extended, but fixed set of points (P, Q, R, S, and T waves) [12,13]. 

**Figure 1:**
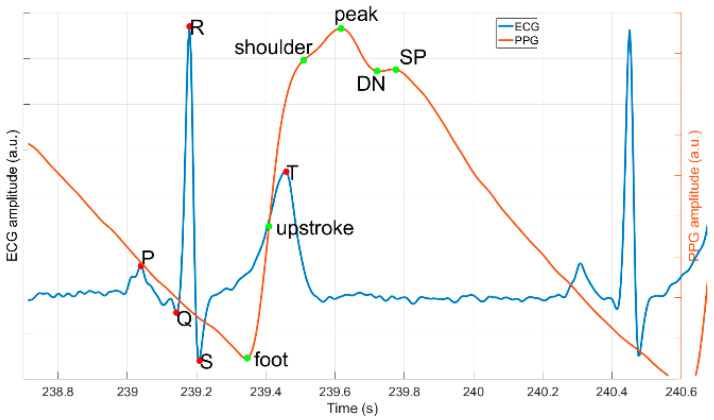
Example signals of one cardiac cycle with corresponding fiducials: P, Q, R, S and T waves’ peaks for electrocardiogram (ECG) (blue); and foot, upstroke, shoulder, peak, dicrotic notch (DN), and secondary peak (SP) for photoplethysmogram (PPG) (red).

The focus in both literature and commercially available medical tools and data on the annotation of ECG signals (electrical activity of the heart) is understandable, since ECG is the standard screening method for diagnosis of many cardiovascular conditions and the most often collected data stream related to heart disease. Clinically relevant information is extracted from amplitudes and intervals of fiducial points of ECG waveform (Figure 1) [16]. Namely, the R-peak located within the QRS complex is of major importance for heart rate (HR) and heart rate variability (HRV) analysis [17]. As a consequence, many R-peak detection algorithms exist based on different approaches—digital filters, derivatives, and wavelets [18,19,20], as well as, more recently, machine learning and neural networks [21]. Kohler et al. [22] published an extensive review of the approaches proposed over the last decades.

Another often-used physiological time series is the PPG signal, an optical-based method that captures volumetric change of blood vessels over time. PPG beat-to-beat intervals correlate closely to the ECG intervals, as they belong to the same cardiac cycle, therefore, PPG modality is considered an ECG surrogate for HR [23] and HRV [24,25,26] extraction, but can also provide additional information [27,28]. Over the last years, PPG has gained a lot of attraction due to its higher wearability. However, that comes with the cost of new signal processing challenges: the PPG signal is very sensitive to motion. In addition, the morphology of the PPG pulse is very different from that of the ECG (Figure 1), hence, different algorithms are needed for processing of the PPG signal. The pool of mature PPG algorithms is sparser compared to ECG [29,30,31,32], and this is partially due to lack of proper public benchmarking datasets.

One of the most important sources of labeled clinical signals is the Physionet platform [33], which includes dozens of ECG datasets aggregating to millions of ECG beats. The MIT–BIH dataset [34] is a dataset within the Physionet platform and has become the de-facto standard for QRS detectors validation, because of its versatile set of waveforms with clinically significant arrythmias and annotated R-peaks. Even international medical standards for ECG processing [35] use the MIT–BIH dataset as a reference.

In contrast, for many other physiological time series, such a large, annotated reference dataset does not exist. Indeed, public PPG datasets are scarcer, and published algorithms report validation results on in-house recorded small-scale databases with less than 10 subjects [36].

The Platform for Analysis and Labeling of Medical time Series (PALMS), a flexible tool that meets the requirements listed above, was made to enable the creation of such benchmarking datasets for algorithm development. In this paper, we present the platform, a fusion of a GUI and signal-processing algorithms, providing means for (optional) automatic detection, display, and manual correction of reference information (annotations) to biomedical time-series data. In the rest of the document, PALMS functionality, GUI, and algorithms are demonstrated on two of the most often used physiological signals: ECG and PPG. The PALMS ECG algorithm is a wavelet-based method for reliable R-peaks identification. The PALMS PPG algorithm is a time-domain fiducial point detector based on the derivative of the PPG. It detects multiple pulse fiducials’ locations per heartbeat, including a maximum peak amplitude, a time of a foot (onset), and an upstroke (Figure 1). In addition to the description of PALMS, this paper presents validation results of the two algorithms and discusses the applicability of the framework.

## 2. Materials and Methods

The design and functionality of PALMS including a role-based system is explained below. It describes the role-based control system, the user interface, the configuration process, and the two fiducial point algorithms.

**Figure 2:**
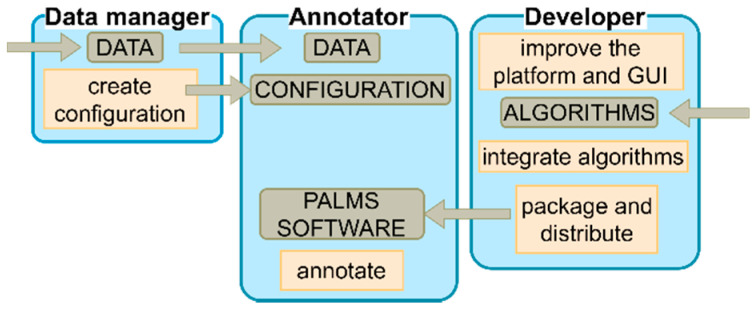
Different roles within the Platform for Analysis and Labeling of Medical time Series (PALMS), for each role the responsibility is depicted in the orange boxes, required information in gray boxes, and the information direction is given by the arrows. GUI—graphical user interface.

PALMS has a role-based design that defines the responsibility and functionality of each of the three roles, namely, the developer, the data manager, and the annotator (Figure 2). PALMS developers improve the platform, integrate algorithms, and make the tool available to users. Data managers (advanced users) are responsible for setting up PALMS for annotators by following well-defined steps (i.e., creating a configuration file). Finally, annotators (e.g., clinical specialists) only intend to annotate data and, hence, need to have access to the data, configuration, and PALMS software.

### 2.1. PALMS User Interface

**Figure 3:**
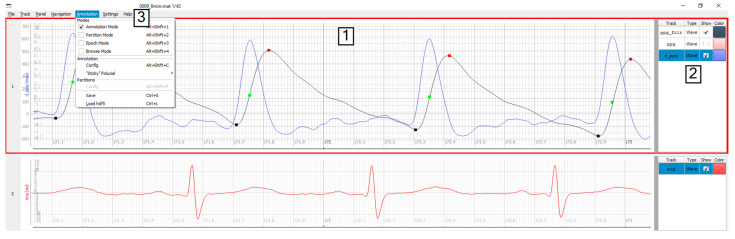
Main window: plot area (1) is split into two panels, plot controls (2) displaying three tracks, and the menu (3).

The GUI used by annotators was developed in Python 3 using an open-source software project “timeview” [37] distributed under the MIT license as a backbone for creating the main window style. The main window (Figure 3) is split into three sections: (1) plot area, (2) plot-controls, and (3) the menu bar.

The plot area, where timeseries data and annotations are visualized, occupies the largest part of the window. There is no limit on how many signals can be shown simultaneously in the plot area. Alternatively, to comfortably observe three or more waveforms, it is possible to split the plot area into several synchronized display panels. In Figure 3, for example, the PPG derivative (blue) and the ECG (red) signals serve as references for the PPG (black).

The plot-controls area on the right of each display panel contains options for changing the visibility and appearance of the plots. A submenu can be reached by a right mouse click in the controls area and this menu allows to plot additional signals (e.g., derivatives, filtered versions) of any original wave. This feature is helpful when annotating fiducials like PPG upstroke, which are defined by the local maximum of the derivative of the PPG signal. In addition, inter-beat intervals of any fiducial can be added to the plot to instantly see possible false positive or false negative annotation locations, visible by spikes or dips in the heart rate.

In the menu bar annotators can find tabs with specified set of commands: File—for restarting the tool, switching to the previous or the next record; Track and Panel tabs—for controlling the appearance of corresponding objects; the Navigation commands (alternative to the mouse wheel and keyboard) allow continuous browsing through signals without delays; the tool documentation and the list of shortcuts is accessible under the Help tab; and primary functionality controls are located in the Annotation menu: (1) Save and Load action buttons export all workspace data to the hard drive and load the data for (re)editing, and (2) mode selection dialog switches the tool into one of three modes—annotation, partition, or epoch mode. 

The default annotation mode is used to register instantaneous events, which have no duration. It is well suited for labeling several fiducials (R-peak, T-wave, etc.). By adding an additional label close to the regular annotations one can indicate the fiducial’s specific property or type, e.g., R-peak of an ectopic, irregular beat (see also the partition mode for the same purpose). In Figure 3, three fiducials (square markers) are annotated simultaneously for a single PPG signal: foot (black), upstroke (green), and peak (red). The mouse is used as the main control unit with left and right buttons configured to place a new or to delete a label, respectively. To distinguish between label types, preset keyboard keys should be pressed before the click. Annotation settings can be initialized in the database configuration file (see B. PALMS Configuration), but are accessible from the GUI too. PALMS implements several features to streamline the process of labeling, such as a user-defined minimum distance between two labels and/or automatic pinning of an event timestamp to the nearest peak or valley regardless of the exact position the annotator has clicked.

**Figure 4:**
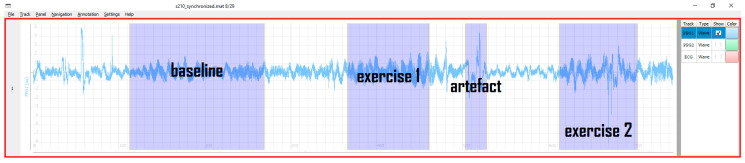
Example PPG signal in partition mode: one can label activities, motion artefacts, and other regions of interest.

Contrary to the annotation mode where events belong to a single timestamp, the partition mode is designed to register events with a certain duration, like, e.g., artefacts, interventions, or other segments of explicit interest. In this case, each partition possesses a start and end timestamp and a name—all defined at runtime. Partitions may have arbitrary names and durations. After switching the mode, mouse clicks also change their functionality to create, delete, or move partitions. An example of multi-partition annotation is shown in Figure 4. 

**Figure 5:**
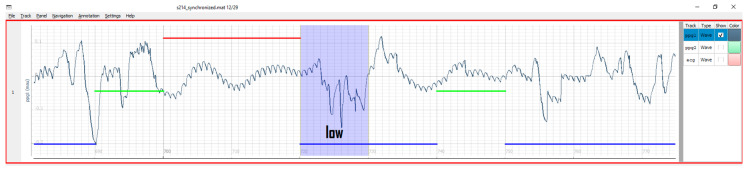
Example PPG signal in epoch mode: blue, green, and red color bars stand for different signal quality levels assigned to 10 s segments.

The main goal of the epoch mode in PALMS is to facilitate quality annotation. Such datasets are rare and can form a valuable training or validation set for creating signal quality indicators or artefact detectors. Figure 5 visualizes a PPG signal of which 10 s chunks can be binned into three quality levels—low (blue), acceptable (green), and high (red). Quality labels and the epoch length can be adjusted in configuration. The labeling is done using the keyboard left/right arrows for navigation and up/down for assigning the level. The epoch mode can also be used for event annotation in applications such as electroencephalography (EEG), where conventionally each 30-s epoch is labelled depending on the neural responses present in the signal.

### 2.2. PALMS Configuration

Prior to labeling a dataset, a configuration file should be created in which a new instance of the Database class is described. The configuration file is made by the data-manager. It encapsulates information about the source and the operations. The class contains several methods (get data, set annotations, save, and load) and fields describing dataset attributes such as source and output folders or filename template. Most of the fields in the Database class have default values, and a number of built-in automatic tests guide data managers through the configuration procedure.

The method get data is triggered when an annotator starts the GUI and selects a recording from a database in a pop-up window. For every new dataset, this method needs to be defined by the data manager as it is the only one without a default (inherited) implementation. Inside get data, the data structure of the corresponding database is “explained” and arbitrary preprocessing steps, e.g., filtering, can be introduced. Execution of get data results in all relevant time-series signals being packaged into Track objects (see C. PALMS Architecture).

Next, set annotations is executed in the background. Its implementation can be left blank, alternatively, via this method it is possible to add prior knowledge about annotations to avoid labeling from scratch. For example, as implemented in the current version of PALMS, we integrated ECG and PPG fiducial detectors within the annotation tool. Those algorithms are then run by set annotations on matching signals, which then stores outputs in corresponding Annotation class instances. Alternatively, it can contain interface code to load existing annotation files (e.g., Physionet standard) and bind them to raw data. Such code would be reusable for any database of that kind.

After these automated procedures, the user can browse through time-series data and manually create (edit) annotations. Using default save and load methods, the workspace can be stored (in HDF5 format [38]) or loaded for reediting at any moment. PALMS is provided together with Python and MATLAB scripts for opening of output. h5 files.

The overhead from configuration procedure is limited as it has to be done once per dataset and the file can be reused by all annotators, whereas customizable configuration removes restrictions on input data format and underlying preprocessing steps, and thereby creates the flexibility required to use PALMS for a variety of applications.

### 2.3. PALMS Architecture

The software was designed in Python using object-oriented style with three top-level classes—Database, Track, and Annotation. Annotation objects store information about fiducials, partitions, and epochs belonging to one of the signals (Tracks). User interaction with annotations is implicit: corresponding objects are updated as soon as user makes changes in the data via the GUI. Each Track object represents a signal in the dataset in terms of sampling rate, amplitude values, time offset, etc. Annotators can control which tracks are displayed and infer new tracks (e.g., time derivative) from existing ones. All tracks together form a Database object, which is an interface between PALMS and arbitrary annotation project. Each Database is described by dataset managers using configuration file templates. The PALMS source code remains independent from configuration and does not have to be changed. Moreover, developers can create a portable executable version of PALMS and distribute it among annotators, which allows them to use PALMS without having to change or even access the source code. Python environment installation is also not required: users need to run the stand-alone PALMS executable placed in the same folder as configuration files.

### 2.4. PALMS ECG Fiducial Detector

**Figure 6:**
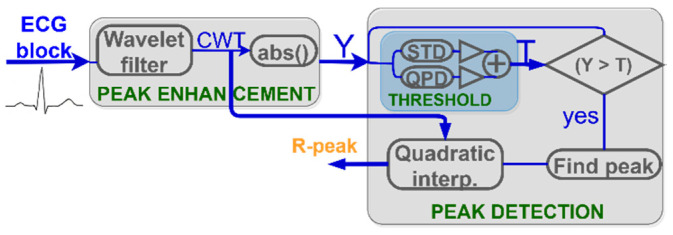
PALMS ECG algorithm block diagram: CWT (continuous wavelet transformation), Y (absolute value of CWT), STD (standard deviation), QPD (quasi-peak detector), and T (threshold).

The QRS complex is the most prominent part in the ECG waveform and is exploited in heartbeat detection algorithms [22]. The block-diagram of the R-peak detection algorithm incorporated into PALMS is shown in Figure 6. The detection process is split into two stages—peak enhancement and peak detection—each with several sub-steps. All operations are done sequentially on the ECG blocks of 0.5 s duration. A special state variable maintains information transfer from the previous block to the next in order to detect heartbeats split between two or three blocks. This significantly reduces the required buffer size and removes the need for processing with overlapping segments.

**Figure 7:**
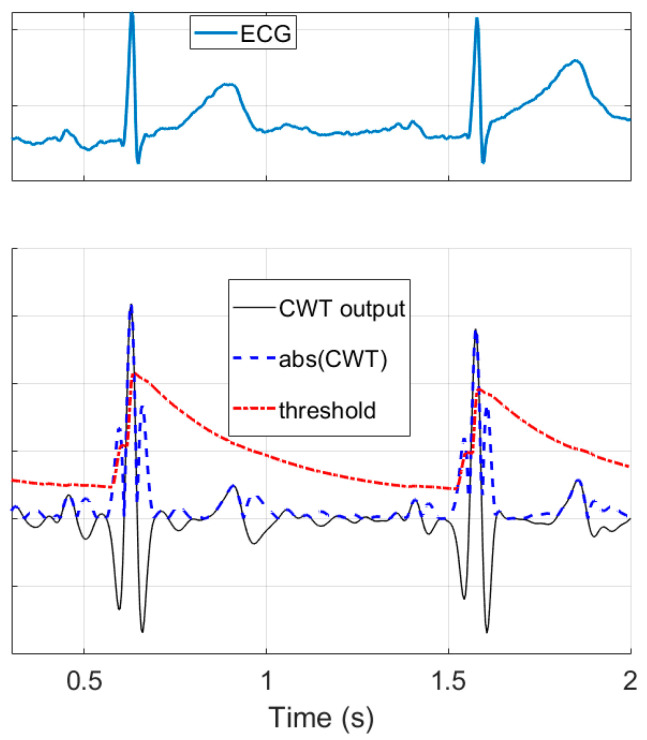
Example of the ECG wavelet transform with the following threshold generation: original ECG (top), wavelet transformation (CWT, bottom, black), absolute value of the CWT signal (bottom, dashed blue), and detection threshold (bottom, red).

The algorithm employs a similar approach as Romero et al. [39], with several modifications. After R-peak enhancement by the discretized continuous wavelet transformation (CWT) and applying the modulus maxima operation (Figure 7), the signal Y is compared to the threshold. In contrast to Romero et al. where a universal preset threshold of 30% of the maximum value was used, PALMS ECG recalculates the threshold for each signal block value (blue block in Figure 6) based on the weighted sum of the noise and signal level. The noise level is calculated as the running average of the standard deviation (STD) of each data block. This prevents false detections in the presence of noise. The signal level is calculated by a quasi-peak detector (QPD), a 1st order IIR filter with a bandwidth that reacts to the difference of the current and the previous samples. Noise level and QPD output are summed and scaled to form the threshold value. Scaling is done to balance between detecting true early peaks (premature beats or atrial fibrillation) and misdetecting high amplitude T-waves. 

Another adjustment to Romero et al. concerns precise R-peak location being now refined by quadratic interpolation method—fitting a second-order polynomial across the detected maximum and two adjacent samples. An exact R-peak location is calculated to allow intra-sample precision at the maximum of the fitted curve.

### 2.5. PALMS PPG Fiducial Detector

PPG fiducial detection algorithm included into PALMS is based on the first derivative with the idea being similar to Li et al. [40]. Authors focused on arterial blood pressure (ABP) signals to detect systolic peak, onset (foot), and dicrotic notch (DN) locations. PALMS PPG benefits from the fact that ABP signals are morphologically analogous to PPG, but extra decision logic is introduced to detect more fiducials: the upstroke, secondary peak (SP), and shoulder (SH).

**Figure 8:**
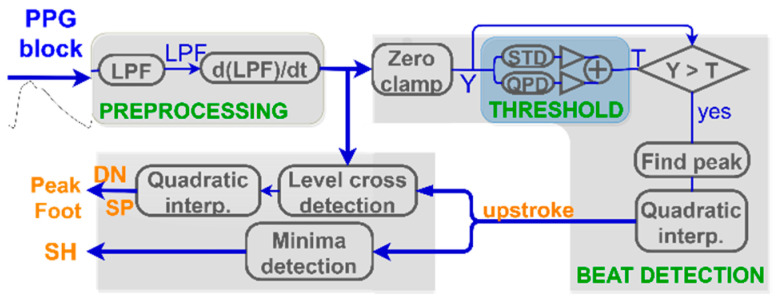
PPG algorithm’s block diagram: each PPG block is processed to find locations of characteristic points (orange): LPF (low-pass filter), Y (zero-clamped LPF), STD (standard deviation), QPD (quasi-peak detector), and T (threshold).

In a nutshell, the proposed algorithm uses the upstroke detection as starting operation, which is split into preprocessing and beat detection stages followed by an additional block responsible for derivation of other fiducials as is shown in the block-diagram at Figure 8. At the first step, data are band-pass filtered by a combination of a low pass filter (LPF) with the default cut-off at 10 Hz and a differentiator. The latter outputs a differentiated (dLPF) signal that is used in the beat detection stage, at which fiducials’ locations (and, subsequently, amplitudes) are estimated.

The main fiducial point to be extracted is the upstroke being the local maximum of the dLPF. As the polarity of the upstroke is always positive, we apply a zero-clamp block to force all negative values to zero. The output of the operation is abs_dLPFS, further used in the custom peak finding function. The function implements a state machine with adaptive amplitude threshold and previous state preservation analog to the procedures used in the ECG beat detector.

**Figure 9:**
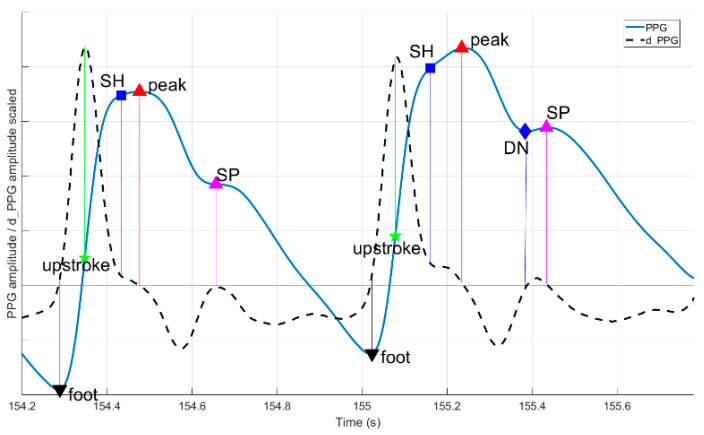
PPG algorithm fiducials’ definition: every beat must have foot, upstroke, and peak, other fiducials may be present depending on exact waveform. DN—dicrotic notch, SH—shoulder, SP—secondary peak.

If the upstroke location was found, other fiducials are estimated as indicated in Figure 9. Foot and systolic peak are defined as the last zero-crossing before and first downwards oriented zero-crossing after the upstroke, respectively. Only if upstroke, systolic peak, and foot are found, the beat is valid and the algorithm proceeds with finding secondary fiducials: SH, SP, and DN. It checks for a shoulder presence as the first trough in derivative between the upstroke and the peak. For SP and DN, a search time and amplitude windows are defined as proportions of foot-to-peak rise-time and amplitude change. Both SP and DN are present if additional abs dLPFS zero-crossings are discovered within the time limits given as parameters. If some fiducials are not found, their values are set to not-a-number. Using fiducials’ timestamps, multiple features are calculated inside the algorithm: rise and decay times, amplitudes, and their ratios.

### 2.6. Algorithms’ Validation

The value of fiducial point detectors within PALMS depends on the accuracy of the algorithms, therefore, both algorithms were validated using public annotated datasets and in-house labeling done by the authors.

During development, the optimal set of parameters was defined using multiple databases with more than two million beats in total. In addition to several publicly available datasets (the MIT-BIH and the European ST-T Database), we also used in-house ECG data from healthy volunteers doing different activities, and datasets collected at Ziekenhuis Oost-Limburg (Genk, Belgium) from patients included in a rehabilitation program and patients admitted to a sleep clinic, and datasets collected at Maastricht University (Maastricht, the Netherlands) from healthy population during daily activities [41,42,43]. To include underrepresented morphologies, we simulated examples of ECG with varying QRS width, QT intervals, and HR using the ECGSYN [33] software package.

The ECG algorithm was tested on the full MIT–BIH database (48 half-hour recordings with a total number of 109,494 beats). All estimated beats were binned into true positives (TP), false positives, and false negatives. To assess TP, the value of error tolerance τ needs to be defined, i.e., the maximum allowed time between the true (annotated) R-peak and the detected R-peak by the PALMS ECG fiducial detector. The value of the error tolerance is a major determinant of the F1 score and the error rate. In many publications, the value τ is not reported. Therefore, we used two: 50 ms and 125 ms [19]. The reasoning is that 50 ms is about half of the normal QRS complex width, and 125 ms—corresponds to the half of the highest physiological HR of 240 bpm. Because the number of true negatives cannot be defined, performance was quantified by the F1 score [44] and error rate as the ratio of false detections and TP.

We evaluated the performance of PALMS PPG on two publicly available databases (BIDMC PPG and respiration dataset [45] and IEEE TBME respiratory benchmark dataset [15]). Datasets contained 53 and 42 8-min recordings, respectively. Assuming PPG waveshape uniformity within each recording and diversity between recordings, we extracted one 30-s segment from each recording. Then foot, upstroke, and peak of every PPG beat was labeled from scratch taking into account that the reference ECG signal labeling was done by one annotator with experience in biomedical engineering and PPG annotations. For quantifying performance, we used an approach similar to ECG with τ = 50 ms.

### 2.7. Dataset Labeling Example

To illustrate the potential benefit of using PALMS, we evaluated the annotation of a dataset from the Physionet Computing in Cardiology Challenge 2011 [33] with quality annotations (see Figure 5). Originally the dataset was annotated with two quality levels. For more advanced algorithm development, the data was reannotated with 5 different labels (Table 1) resulting from two-fold evaluation: each ECG trace of 10 s could get Q0 (low), Q1 (acceptable), or Q2 (high) for quality score, and M0 (low) or M1 (high) for morphology score with definitions as in Table 2. Recordings were annotated by four experts, and each recording was labeled by at least three experts. In case of disagreement among them, recordings were first reviewed by the fourth reviewer and then, if no 3-to-1 majority reached, by the whole group of annotators together to assign a definite label.

PALMS configuration and annotation rules for this task are available as one of the examples together with the source code. The task can be repeated after downloading the Physionet dataset to a local folder.

## 3. Results

In this work, we presented PALMS—a novel software solution for annotating time-series data. Figure 3, Figure 4 and Figure 5 show the visual appearance of PALMS GUI. It is built in open-source Python, can be packaged into one file, and distributed without any additional software and the source code, which makes primary functionality (annotation) available even to non-technical users. At the same time, a well-designed configuration procedure allows PALMS to work with various formats and data sources, thus the annotation capabilities are not restricted to a particular signal type. Furthermore, annotations themselves can be of three types: zero-duration fiducials (timestamp and amplitude information), time events in which the start and end time are annotated (partitions), and epoch that allows scoring of signal quality or other features for a prescribed time window. Multiple fiducials can be simultaneously annotated in the same signal, e.g., it is possible to mark locations of R-peaks and T-waves of the ECG or systolic and diastolic peaks of the PPG at the same time. 

To further assist human annotators, PALMS provides a semi-automatic labeling option: advanced users (developers) of the platform can integrate custom algorithms with minimum effort. Currently, the platform contains fiducial detectors for both ECG and PPG signals to promote faster processing of physiological data. PALMS ECG isolated validation showed an F1 score of 99% for τ = 50 ms and 99.5% for τ = 125 ms, the error rate of 2% and 1% correspondingly. Visual inspection reveals that algorithmic errors are often related to abnormal and ectopic beats. PPG algorithm validation results for each fiducial and database shown separately in Table 3.

In addition, we provided a demonstration of PALMS by adding a set of new labels for signal quality with custom rules to enhance an existing public dataset. New annotators found the tool intuitive to work with and enabling fast and accurate annotation. In particular, labeling of about 40% of ECG segments from the Physionet 2011 Challenge took less than 5 s per segment. It can be said that PALMS operational delays due to data loading, visualization, and saving of results are negligible to the time experts need to observe the data and make a decision.

## 4. Discussion

The performance of PALMS was evaluated in several ways. Isolated PALMS ECG performance is high and similar to other ECG fiducial point detector in literature. The well-known Pan et al. algorithm [46] reported an error rate of 0.7%, although higher than PALMS ECG on specific recordings. The Li et al. [47] algorithm resolved the same database with 99.9% sensitivity and predictive value of 99.94%, but high accuracy is partially achieved by introducing a post-processing step, during which redundant detections are discarded. The Romero et al. [39] algorithm is similar in design and reported results in sensitivity and predictivity above 99.6%. Since not all studies used a full version of the MIT–BIH and few reported detection error tolerance value, direct comparison is not always valid.

Future development should further investigate PALMS ECG performance for abnormal beats to tell if any particular abnormalities (large T-wave, broad QRS, irregular HR, or else) cause systematic misdetection. Automatic parameter optimization would allow to deal with some abnormalities on case-by-case basis.

The performance of PALMS PPG cannot be compared to other algorithms because they make use of different datasets. Luo et al. [32] presented a beat onset and peak detection framework based on prior estimation of baseline and the power spectrum, which scored 99% accuracy on 100 short PPG segments. Tran et al. [48] proposed a combination of an adaptive peak detection threshold and the “random error method” and reported a failed detection rate of 1.1%. Our validation shows slight variation in scores between foot, peak, and upstroke with minor advantage of the latter. This may come from the fact that PPG extremums are more prone to noise than steep rising edge, where the upstroke is located. This implies upstroke is potentially a better choice for PPG beat detection if millisecond-level precision is important.

PALMS PPG was validated on two datasets acquired in rest conditions. Although, high performance was reached, such validation does not cover some applications, e.g., HR tracking during sports. A post-processing block to revise detected points once full information is available also could improve the performance.

The current version of PALMS already has large flexibility and conforms to the requirements stated in the beginning, but further developments could even widen its applicability. Future updates may bring functionality to annotate multiple signals at once, e.g., for multi-channel ExG signals. Another useful features may be an automatic comparison and merging of several annotation files done by different experts. To promote PALMS usage with more novel datasets, it should be accompanied with corresponding algorithms. For instance, motion- (accelerometer) or respiration-related algorithms would be easy to build into PALMS as black boxes. They would provide a good initial guess for annotations, which, after manual corrections, could be used to develop improved algorithms’ versions. 

## 5. Conclusions

PALMS is a Python-based GUI for rapid annotation of biomedical time-series datasets. The main advantage of the platform is the ability to integrate annotation algorithms and its high level of customization. Its role-based design allows discrimination between clinical annotators (ignorant of the underlying object-oriented design), data-managers (can configure PALMS for a specific database and application), and developers who can use this tool to develop and improve their algorithms. Annotators can assign labels of three types (fiducials, events, and signal quality) simultaneously to one signal. Beyond the two cardiovascular signals, ECG and PPG, analyzed in this paper, PALMS accepts other types of data from different sources, e.g., bio-impedance signals for respiratory monitoring, ExG from brain or muscles, accelerometer from gait, and more. However, the potential benefit of PALMS is not limited to the biomedical domain, it could be applied in any field where domain expert knowledge is required for classification and labeling of time series data. To the best of our knowledge, none of the existing software solutions provide comparable functionality. 

PALMS GUI is available free of charge under the GNU General Public License (GNU GPLv3+) license and can be downloaded from https://github.com/PALMS-gui/PALMS. Demo videos are available at https://vimeo.com/user129053181.

## Figures and Tables

**Table 1:** Annotation labels.

Label	Code	Explanation
0	Q0	Bad quality
1	Q1 M0	Acceptable quality, not good morphology
2	Q1 M1	Acceptable quality, good morphology
3	Q2 M0	Good quality, not good morphology
4	Q2 M1	Good quality, good morphology

**Table 2:** ECG annotation conventions.

Signal Quality	
Q0	<80% of QRS complexes are undoubtfully identified, reliable heart rate (HR) calculation not possible
Q1	≥80% QRS complexes are undoubtfully identified, HR calculation is credible
Q2	Q1 and no R-peak position uncertainty, no artifact, heart rate variability (HRV) calculation is possible
**Signal Morphology**	
M1	P- and T-wave can be seen in nearly all QRS complexes
M0	Not M1

**Table 3:** PALMS PPG validation results (F1 score).

	TBME ^1^	BIDMC ^2^
Foot	94.6%	96.8%
Upstroke	98.6%	98%
Peak	97.6%	96.8%

^1^ IEEE TBME respiratory benchmark dataset [15]. ^2^ BIDMC PPG and respiration dataset [45]
